# Notes on Medical Matters and Medical Men in Paris

**Published:** 1848-05

**Authors:** David W. Yandell

**Affiliations:** Louisville, Kentucky; Paris


Art. III.—*Notes on Medical Matters and Medical Men in Paris.* By David W. Yandell, M.D., of Louisville, Ky.

I am gratified to be enabled to present to the readers of the Journal the Lectures of Professor Dubois, on Hemorrhage in the last three months of pregnancy, delivered during the past month, at the *Hopital des Cliniques.* They number four in all, two of which I shall embody in this, and the remaining two in my next letter.

Since the beginning of December last, five women in our wards have offered uterine hemorrhage, occurring at an advanced period of pregnancy, with the exception of one case, in which the accident came on at about the fifth month of gestation. In some of these cases hemorrhage was renewed during labor. In the case of three women it may be attributed to an *accidental* cause. In the first to a fall on going up stairs, the abdomen coming in contact with a step. In the second to a violent paroxysm of coughing. In the third to a violent emotion. In the two remaining cases the hemorrhage was owing to the insertion of the placenta over the internal orifice of the cervix.

We have therefore to study three cases of *accidental* hemorrhage, and two cases of spontaneous or *unavoidable* hemorrhage. Let the cause have been what it may, accidental or spontaneous, there remains a constant fact throughout all the cases, viz: that in every one there has existed a separation of the placenta. We can therefore advance that *loosening of the placenta* has, in all these cases, been the *proximate* cause of the hemorrhage.

One of the females was a primipara; the four others were pregnant for the second, the third, the fifth, and the eighth time. One of them was brought to bed about the fifth month, two at the seventh and eighth months; and two at eight months and a half. Those with whom hemorrhage was spontaneous were delivered; the first at the eighth month, and the other at eight months and a half.

In most cases the hemorrhage was renewed. In the case of accidental hemorrhage resulting from violent coughing, the flooding did not re-appear during labor; it was also suspended during parturition in the cases of *placenta prævia.*

In all those cases it was an easy matter to ascertain the peculiar cause of the hemorrhage, accidental or spontaneous; this knowledge was acquired by the touch and by questioning the patient. All the five mothers recovered very well; their five offspring were born alive—even the one born at the fifth month, though it is necessary to add that this latter only lived a few hours, for it was not viable. The whole of the five children were examples of head presentation.

The treatment was very simple. For accidental hemorrhage, the woman was kept quiet in bed and given cool acidulated drinks, and this was found sufficient. If hemorrhage did recur in two out of the three cases of this class, it certainly did not present the characteristics of a real flooding, being but a small oozing of blood. In the cases of cervico-placental hemorrhage (placenta prævia) the same treatment was, first of all, resorted to; then, a little later, we had recourse to opening the membranes, which is certainly the best means of saving the life of both mother and child.

Such, said Prof. Dubois, are the cases that we have observed. They bring up all the questions relating to uterine hemorrhage. It is not, however, my intention to investigate the whole of these; but I shall rather confine my remarks to the most prominent points in the history of those hemorrhages which occur during the last three months of pregnancy.

The phenomena we have just noticed as having been recently observed by us belong to the *external* or *apparent* hemorrhages. You will infer from this remark that there exist other kinds of hemorrhage. On this subject allow me to offer a word of explanation. In the five cases we have had under our care, hemorrhage was due to a rupture of the connexions existing between the ovum and the uterus, and the blood was enabled to flow through the genital organs and appear externally. But it might have so happened that the blood would not appear externally; its effusion might have been confined between the uterus and the placenta; under which circumstances it would have been termed *internal hemorrhage.* As this is both an interesting and curious scientific point, and one which you all know has given rise to no small amount of discussion, you will bear with me while I attempt to develope its more important features.

First of all then, hemorrhage following upon the rupture of vascular connexions existing between the uterus and the ovum, can take place inside the placenta itself, in the proper tissue of this organ; it is then called placental apoplexy. On the other hand, the apparatus which unites the infant to its mother may become ruptured in some part of its continuity—at the umbilical cord, for instance, either from the isolated vessels being spread over the membranes, or from a laceration of the funis effected during a manœuvre. Hence will arise various species of internal hemorrhages. They may be all included under the single term *intra uterine;* at the same time, however, they admit of sub-divisions, founded on the peculiarities of the case, into *simple intra uterine; intra placental* or *intra amniotic.*

Professor Dubois after promising to revert to this subject in its proper place, proceeded to treat of the cases which, properly speaking, belong to the present lecture. Before doing so he remarked, that he felt it incumbent on him to meet a consideration which undoubtedly would arise if it had not already arisen, in the minds of his hearers; viz: that in so short a time we should have witnessed so very large a proportion of these hemorrhages; five cases having occurred in the course of five weeks, which is in the proportion of five to one hundred and four cases of delivery; that is, one woman out of twenty delivered has been subject to hemorrhage. You must not believe that such is by any means the ususal course of things. The excess, in this instance, is due to the circumstance of many of these women having been brought to the hospital for the hemorrhage alone, who, had that accident not occurred, would have remained at home to be delivered. This large pro portion, this exalted figure that we have obtained, belongs therefore to a population far more numerous than that formed by the sojourners in this establishment. You find in this fact the reason why good statistics cannot be gathered in special establishments like this hospital, where difficult cases abound, if we compare what passes here and what takes place in ordinary practice.

Of the five cases of hemorrhage that occupy us, the subject of one alone was a primipara. But this fact, however, important might be its signification under other circumstances, possesses but little weight in the present case, for the reason that we have embraced in one group both accidental and spontaneous hemorrhage, and that primiparæ and multiparæ indifferently, are exposed to these accidents. Still it is satisfactorily established by the statistics of Professor Simpson, on this point, that spontaneous hemorrhage is much more frequent in multiparæ than in primiparæ. Thus, out of eighty cases of spontaneous hemorrhage, Professor Simpson has found eight women with child for the first time—that is in the proportion of one primipara to ten multiparæ.

Now with regard to the period of pregnancy at which delivery occurs, the remarks I am about to make, on account of the indiscriminate assemblage of various cases into one class, to which I have already alluded, are intended simply, and they must bear no other signification, to draw your attention to the remarkable coincidence existing between the period at which the two women with *placenta prævia* were delivered, and the period at which are delivered the greater number of women placed in similar conditions. This will be clearly shown by what follows. The two women in our wards laboring under this cause of hemorrhage were delivered, one at eight months, the other at eight months and a half—both, in other terms, at an advanced stage of their gestation. To those results I could add many others, derived from personal observation; but I will content myself at this time with acquainting you with the experience of Professor Simpson on this subject. Out of ninety cases, this gentleman found but three women who were delivered before the sixth month; the others were brought to bed as follows: five from the sixth to the seventh months: nineteen between the seventh and eighth months; nineteen from the eighth to the ninth month, and the forty-three others in the course of the ninth month.

You will remember the remark that I made concerning the late period at which spontaneous hemorrhage generally occurs. Baudelocque and those of his school, taking as a starting point an ill-founded opinion, insisted that this kind of hemorrhage ought not to occur later than the sixth month, or, at latest, the seventh month of pregnancy. This view originated in the idea entertained by those writers, that certain modifications took place at that early period by an incipient dilatation of the uterine orifice. From well authenticated facts we believe ourselves justified in asserting that such is not the fact, but that the lower segment of the uterus and cervix give way to the enlargement of the uterine cavity in the majority of cases, only in the course of the ninth month. This opinion I consider as being far better supported than the one set forth by Baudelocque.

As to the *cause*, we believe ourselves justified in admitting, that in some of the cases hemorrhage came on accidentally, as from a fall, violent coughing, emotions, &c. I will revert one instant to this point on account of the denial by some authors, that the aforesaid causes can induce hemorrhage at an advanced period of gestation. I refer to Mesdames Boivin, Lachapelle, and M. Velpeau. These authorities found their opinion on the fact, that the uterus, in pregnant women, cannot separate that which is contained from that which contains, for the reason that the mechanical action is simultaneously and equally sustained by both bodies. But I beg you will remark, that this is only apparent. When we see, in a fall, the various parts of a body separated by a kind of sliding movement, we must admit that two bodies connected by a third one, of a less solid nature than those it is destined to connect, may be divided, one from the other, by the destruction of the uniting body. In the present case the two bodies are the uterus and the placenta; their connection is effected by means of a vascular tissue which must tear when a violent action depresses the uterine parietes.

Professor Dubois here related a case of a woman who while at the Maternité, had attempted to carry under her arm a folding bed on which there lay a mattrass. Just as she made the effort she felt a sort of cracking sensation in the abdomen which frightened her a good-deal; a very short time afterwards she had a flooding, and it was easy, sometime later, to ascertain that the placenta had been detached at a considerable distance from the orifice.

With all these women, said Professor Dubois, the placenta alone was principally loosened, and this fact may be easily conceived from the following anatomical data. At an early period of pregnancy the ovum adheres to the whole internal surface of the uterine cavity by the medium of general vascular connections. These connections are in every way important as they are called upon to contribute towards the development of the ovum by allowing its drawing from the surrounding surface the elements necessary for its growth.

At a more advanced period of gestation the important vascular connexions become limited to the adherences which are permanent between the placenta and the uterus, in such a manner that the separation of what may still remain of the old general connexions is followed by a slight effusion of blood in ordinary cases; although I do not mean to say that in one particular case, where the vascular element, instead of being atrophied as in the majority of cases it really is, it may not still retain sufficient permeability for the loss of a certain quantity of blood; what I wish to impress on your minds is, that in the great majority of cases abundant hemorrhage implies detachment of the placenta.

Here a question offers itself which sometimes perplexes the mind by the vagueness of the solution generally given to it; namely, which of the two individuals is it, the mother or the child, that loses blood during hemorrhage? From the very facts now under consideration we can in some degree elucidate the matter. Were the children of our five women born alive? Yes; they were all born alive, and we must add, that one out of the number was born alive at the moment its mother was growing cold and pale from the effects of severe hemorrhage; and, moreover, that this same child happened to be the one born under the most unfavorable conditions—that of not being viable. The child in question was born, as I have before said, at the fifth month, but did not live more than a couple of hours. From this fact alone we can easily divine, that the blood lost during hemorrhage, belonged to the mother alone and in no wise to the fœtus. It is from the inner side of the uterus that the blood issues forth, although laceration of the uterine surface of the placenta may also cause a loss of blood to the child. I will ask if, hitherto, in cases where hemorrhage had placed the mothers life in danger, the children have been found deprived of blood? no; certainly not; on the contrary those children always appear full of blood and even congested.

Professor Simpson has adopted the view that the blood issues from the loosened portions of the placenta, and that it takes place without the fœtal existence being endangered. Professor Simpson alludes to the fact of the existence in the placenta of a fœtal portion and a maternal or uterine portion in which the blood of the mother circulates through canals. "Blood," says Professor Simpson, "may be effused out of those canals, without there being any oozing of the blood from the uterine surface; the arteries of which are long and obliterated by laceration, and the opened veins themselves are closed up by a sort of membranous valve." Professor Dubois said that his experience led him to reject this explanation, and he would ask of Professor Simpson the question, whence the blood proceeds in hemorrhage occurring after delivery at a moment when there remains neither fœtus nor placenta *in utero?* If the blood is not furnished by the long, slender, and lacerated arteries, it must be then by the uterine veins; and these uterine sinuses,—those canals which constitute a real cavernous tissue,—whence would they receive their blood, if not from the arteries? I prefer entertaining the opinion that the undetached portion of the placenta keeps up circulation in the uterine parietes not only in that portion of them which corresponds to the adherent portion of the placenta, but also in that other portion from which the placenta has become detached. The blood thus called to the uterine surface, flows in an equable manner towards its whole substance, and is there effused when it is called to the part where the detached placenta can receive it no more. Hence the extravasated blood oozes downwards between the uterine parietes and the ovum, and when sufficient in quantity, appears externally at the genital parts. Thus it is the mother that loses the blood, and it is but in exceptional cases that the blood comes from a solution of continuity in any point whatever of the circulatory apparatus of the child.

In the cases of accidental hemorrhage we have had occasion to observe lately, the appearance of blood did not take place immediately after the action of the determining cause. A few hours elapsed between the accident which produced the separation of the placenta, and the manifestation of the hemorrhage itself. Although this be the rule in cases of this nature, I think proper, nevertheless, to lay the fact before you in order to give an explanation of it. I say, in cases of accidental hemorrhage, it is rare to see the flooding follow immediately the cause which gave rise to it, and I add, that the fact is owing to the placenta being attached more or less in the vicinity of the uterine orifice; for there exists a well marked succession in the phenomena which then occur. The placenta is loosened first of all; then an effusion of blood is effected between the placenta and the uterus; then the effused blood glides down between the uterine parietes and the membranes; finally it passes through the orifice, down the vagina, and makes its appearance at the vulva. You will therefore understand that these phenomena being accomplished in the order in which I have described them, must require a more or less considerable space of time to reach their final manifestation—external hemorrhage—according to the longer or shorter course the blood has to follow. In two of the women who have come under our care, the placenta was situated far from the orifice; with the third one—she who fell on the stairs,—the placenta was not very distant from the os uteri; for this reason hemorrhage soon became apparent in the latter case. The circumstance of the hemorrhage not following immediately the action of the cause which produces it, will again be referred to when we come to speak of the diagnosis of this kind of hemorrhage.

In two of our cases the hemorrhage was at first suspended, and then reappeared during labor; in the third case the hemorrhage was arrested by the simple means we have already alluded to, and it did not again occur. This course is the natural one, and forms the principal characteristic sign between this class of hemorrhages and that designated by Madame Lachapelle *cervico placental hemorrhage.*

Another circumstance worthy of remark is, that the hemorrhage although suspended, nevertheless brought on premature labor; that is, at five months, seven months, and eight months and a half. This is owing to the effusion of coagulated blood between the uterus and the ovum having determined certain changes which have roused the uterine contraction; and it is worthy of being remarked, also, that labor was not induced immediately after the first appearance of the blood. If things did not take place in that manner, it is probably attributable to the effused blood being in those cases insufficient to bring on contraction; or else the uterine parietes having at first proved insensible to the irritation caused by coagula.

If, now, we come to examine what passed in the two cases of cervico-placental hemorrhage, we find that in one case the flooding was often repeated before the end of the eighth month, at which period the woman was delivered; and that in the second case the first appearance of blood took place at eight months and a half, and that this woman only flooded that single time. There certainly is a most remarkable difference between these two cases. It would be natural to think that in one instance labor had been brought on by the abundant and reiterated floodings, while in the second, that gestation had attained a more advanced period by the fact alone of the hemorrhage being moderate; but we must here call in another cause, *uterine excitability*, which was much greater in one case than in the other.

You will remark, that in cervico-placental hemorrhage there are repeated floodings, separated from each other by intervals, during which there is but a slight oozing of blood, more or less marked. This kind of hemorrhage differs, therefore, in its course from accidental hemorrhage, which has been suspended, or at least, only manifested its return, in two cases, by a slight oozing, while in the third case it did not reappear at all. This is owing to the cause of cervico-placental hemorrhage being a permanent one. The modifications undergone by the uterine orifice cause it to withdraw gradually and continually from those points of the placenta to which it adhered, so that the flow of blood is, in some measure, continually kept up.

In the case of *accidental* hemorrhage, on the contrary, the cause is temporary. There is no reason why the flooding being once suspended should be renewed, unless very active circulation or a certian general state of the body should help to overcome the resistance which nature alone, or aided by art, opposes to this kind of hemorrhage. This natural resistance is the general hæmostatic means opposed by nature and favored by art in all cases of hemorrhage. I mean the deposit of coagulated blood at the ends of the divided vessels, and which arrests the ulterior escape of blood. I dwell upon this fact because it cannot occur in cervico-placental hemorrhage. What we have said of the mechanism of the latter will lead us to understand that the action of this hæmostatic means could only prove in similar cases, to be of a temporary, and therefore, of an insufficient nature.

As to the possibility of stopping hemorrhage dependent upon *placenta prævia*, by other means, I shall revert to this point a little later.

These two cases do not suffice, said Professor Dubois, to give you a complete idea of this kind of hemorrhage. Allow me to add some peculiarities drawn from facts of a similar nature. Thus we might have met with a phenomenon which we were not called upon to witness in our two cases, that is the sensation of a sort of cracking noise which precedes the manifestation of hemorrhage. Again it is not impossible that, in certain cases of placenta prævia, the hemorrhage should only occur once,—that the hæmostatic means should be successful in putting an end to the flooding. There are cases in which the edge of the placenta is inserted over the orifice of the uterus, and it may easily be conceived that if, with such a disposition of parts, a clot should obliterate the loosened part of the placenta, dilatation might continue without destroying any further connections. Thirdly, other cases might have offered to your observation of repeated floodings, succeeding one another at very short intervals, during which there continues an oozing of blood, these losses of blood not being followed by uterine contraction, and the patients becoming pale and œdematous. Then you might have witnessed the nervous accidents properly belonging to patients weakened by a considerable loss of blood; you might have seen labor effected during a copious gush of blood. Then again, labor might have been induced by one single flooding, delivery being effected in the midst of a sudden discharge of blood. You might have witnessed the death of the child born in a similar condition, and also the death of the mother a short time after delivery; and last of all, you might have been present at an overwhelming hemorrhage which would have taken off the patient before she had time to be delivered.

Such are the cases you might have been called upon to see, gentlemen, if it had been your lot to attend a greater number of examples of this nature. Such are the elements which are ordained to fill the measure of your instruction, and complete the extent of your experience on this subject.

*Diagnosis.*—The diagnosis of this kind of hemorrhage, easy for the cases which it has been our lot to observe, may, however, include several important points which must be taken into consideration. The first point consists in determining the source of the flooding. The solution to be given to this problem is easily conceived when we know that the blood which appears at the vulva at an advanced stage of pregnancy may have many sources, and be quite foreign to the connection which unites the ovum to the uterus. Thus the blood may come from the internal surface of the external parts of generation, as it has been seen to do in cases of laceration of those varicose veins which lie on the inner side of the labia majora. The rupture of similar vessels may take place along the vaginal parietes also. The loss of blood might equally arise from a pathological state of the cervix.

Professor Dubois here quoted the case of a woman with whom, said the person who described the case in a letter, there existed hemorrhage due to placenta prævia. Professor Dubois found in this case, that the external surface of the cervix offered numerous granulations, adhering to the tissue of the organ itself, and giving to the touch the sensation of placental cotyledons. There existed in this case a cancerous affection of the cervix, which had proved to be the cause of the flooding.

The second point to be examined in order to elucidate the diagnosis, consists in ascertaining whether hemorrhage is due to *accidental causes* or to *placenta prævia.* This question will be answered by the interrogation of the woman and by the touch.

By gathering from the woman the history of the case up to the moment of our investigation, we endeavor to establish the period of pregnancy attained when the hemorrhage first declared itself; and to learn from her if she is able to attribute the flooding to any appreciable cause.

By the touch we endeavor to find out,

1st. Whether there exists any lesion which might be taken for the separation of the placenta from the uterus, and

2d. Whether in cases where the hemorrhage has evidently its source within the uterus, the insertion of the placenta is near to, or far from, the orifice.

In some of the cases which form the subject of these lectures, we have been able by the *touch* to ascertain that the placenta was not present over the orifice, by the fact that the finger introduced into the os uteri met with membranes and sometimes with the head of the fœtus; moreover, in one case we were able to feel the sutures and the fontanelles through the thin, distended lower segment of the uterus. On the other hand, in one case out of those which now claim our attention, the placenta itself was felt seated inside the cervix; and in the other case we have found, at the orifice itself, a certain thickness of membranes and a peculiar spongy state of the part examined, which if they did not constitute a positive sign of the presence of the placenta, were, however, strongly indicative of it.

But now let us resume each of the elements of the problem, in order to appreciate their full signification. To return to the interrogation of the patient: why is it that we endeavor to learn the precise moment of the manifestation of the hemorrhage? Because it is at a period of pregnancy pretty well determined that we see occur the floodings due to placenta prævia.

Now that this element of the question is found in all cases, we cannot say is positively true, for we should be contradicted by two out of our three cases of accidental hemorrhage, in which the flooding took place at a period when it generally appears in cases dependent upon placenta prævia.

Then, again, why do we endeavor to ascertain, from the woman, if any cause easily appreciated by herself, has produced the hemorrhage, if it is not to elucidate the history of the case by the addition of this other fact, that the flooding appears to be spontaneous in cases of placenta prævia?

Now can the touch throw light upon all these questions? Unfortunately we do not find it capable of doing so. First of all, in primiparæ, the os uteri remains closed up until near the term of pregnancy, and allows, nevertheless, a considerable quantity of blood to ooze through, without the finger being able to detect the presence of the placenta over the lower segment of the uterus. The diagnosis will be rendered more difficult still, if, desirous to judge of the thickness of the membranes or of the lower segment of the uterus itself, we find neither the head nor any other prominent and solid part of the fœtus, to serve as a point of contact by which, owing to the propulsion of the finger, we can measure the thickness of the intervening parts.

Another circumstance which may sometimes offer a temporary but real difficulty in the diagnosis, is the presence of a clot of blood in the cervix. At the moment we present the finger to ascertain the nature of the hemorrhage we find the flooding ceased and the os uteri closed up by clots. It is in similar circumstances, we must learn to yield to a natural effort, and that we must know how to temporize in presence of a plug made by nature. It has been said if blood flows in greater quantity during a contraction of the uterus, that it will be a sign of cervico-placental hemorrhage; this preconceived idea, which would seem to mean that, in such cases, the hemorrhage had its source in the placenta more or less compressed by the contracting uterus, is by no means borne out by experience. There is no doubt but that the contraction of the womb will squeeze out the blood with more vehemence, but that phenomenon will take place let the blood proceed from whatever source it may, so long as it comes under the action of the uterus.

I have passed now in review the difficulties of the diagnosis, though it must be confessed those difficulties are of an exceptional nature, and one may, with prudence, care, and experience nearly always arrive at the knowledge of a placenta placed over the os uteri, but not so frequently when the placenta is grafted into some of the neighboring points of the uterus.

The result, I have said, has been fortunate both for the mothers and their children. But on this score we must not be led into erroneous opinions by the cases observed in our wards;—first, because those cases are not numerous; secondly, because in this limited number of cases we have three of accidental hemorrhage, and these are seldom of a serious nature; thirdly, because in one of the cases of placenta prævia the figure of the placenta was such as to allow its detachment to be effected principally in a point where there existed a membranous expansion between the vascular portion, which was indented like the figure of a heart. In this singular case, therefore, very few vessels have been lacerated during dilatation, and the hemorrhage may have been much less serious owing to the anatomical disposition of the placenta; fourthly, because those cases happened in our wards at a moment when the sanitary condition of the hospital was in every respect satisfactory. At first sight of the matter one might be inclined to think that hemorrhage occuring at the moment of labor ought to obviate or at least diminish the danger of ulterior inflammation. But it is not thus that things terminate; the contrary, even, is the general law. The women who have lost a great quantity of blood are far more exposed than others to fall ill during the puerperal state, and with less chance of recovery, as they are already brought very low by the loss of blood, and unable to sustain, to any extent, the antiphlogistic treatment. In short, those women are the most liable of all, to fall victims to puerperal fever.

You might also be led to believe from what you have recently seen at the *Clinique*, that we have had to deal with accidents of minor importance. Be persuaded to the contrary, gentlemen; hemorrhage occurring in the last period of pregnancy is the most serious, the most dangerous accident that our profession ever encounters. Thus in 389 cases of hemorrhage collected by Professor Simpson, there occurred 133 deaths, or one woman out of three. The average of deaths amongst the children of these women is also one out of three. These, you will remark, are results equal to those found in the gravest diseases. Death, during or after hemorrhage, may be due to various causes:

1st. *Repeated flooding*. 2d. *Obstetric operations*, rendered necessary by the accident and practiced with the view of saving the mother or the child. 3d. *Derangement produced in the placento-fœtal circulation by the child itself.*

I must remark, as relates to this latter cause of death, that it is not indifferent for the child, whether the placenta should be placed in the lower segment of the uterus, or higher up in the cavity of the organ. The body of the child which has a tendency to occupy the declivity of the womb, presses, principally, during the standing position on the lower segment of the uterus and on the placenta when the latter is inserted in that region of the uterus. The placento-fœtal circulation must be more or less deranged by the compression exercised upon it. If you question a number of pregnant women, you will learn that in certain attitudes, during decubitus, for instance, the fœtus becomes at times so turbulent that they feel obliged to change their position. There are many reasons to believe that this or the other position only becomes uncomfortable to the fœtus when there is some derangement in the fœto-placental circulation.

The children who were born of the two women which presented examples of placenta prævia, offered head presentations. This is a rare occurrence in cases of this nature, for the insertion of the placenta over the orifice is a predisposing cause to abnormal presentations. Thus, Professor Simpson, out of 91 cases has found 21 trunk presentations. This may be attributed to two causes; 1st, the children are often born in a state of putrefaction, on account of death produced by the compression just alluded to, and in that case the presentation is nearly always abnormal: 2d, in order that a child may pass through the brim, it is necessary, first of all, that the inferior segment of the uterus should begin by placing itself in the brim, and then the concavity of that segment so placed receives the vertex, &c.; but when the placenta occupies the lower portion of the uterus, the head of the child has no tendency to rest on a filled up or flattened concavity. Hence occurs what happens in a case of malformed pelvis, in which the head cannot place itself with ease, but turns away from the obstacle, advances with greater facility on one side of the upper pelvis, and by that manœuvre, a presentation of the extremity of the ovoid is converted into one of the lateral region, right or left of the fœtus.

This letter ought to have gone to you by the last steamer, but the political commotion, of which Paris has just been the theatre, arrested the mails and I was unable to get it off in time. The revolution appears to be complete, and all for the present is order and peace. A week ago Louis Phillippe abdicated the throne; Paris was never more profoundly tranquil than to-day.

Paris, March 1st., 1848.

